# Tracking the Time Course of Bayesian Inference With Event-Related Potentials:A Study Using the Central Cue Posner Paradigm

**DOI:** 10.3389/fpsyg.2019.01424

**Published:** 2019-06-19

**Authors:** Carlos M. Gómez, Antonio Arjona, Francesco Donnarumma, Domenico Maisto, Elena I. Rodríguez-Martínez, Giovanni Pezzulo

**Affiliations:** ^1^Human Psychobiology Lab, Department of Experimental Psychology, University of Seville, Seville, Spain; ^2^Institute of Cognitive Sciences and Technologies, National Research Council, Rome, Italy; ^3^Institute for High Performance Computing and Networking, National Research Council, Naples, Italy; ^4^Department of Psychology, Loyola Andalucía University, Seville, Spain

**Keywords:** predictive coding, Bayesian processing, ERPs, P300, Contingent Negative Variation, Central Cue Posner Paradigm

## Abstract

In this study, we asked whether the event-related potentials associated to cue and target stimuli of a Central Cue Posner Paradigm (CCPP) may encode key parameters of Bayesian inference – prior expectation and surprise – on a trial-by-trial basis. Thirty-two EEG channel were recorded in a sample of 19 young adult subjects while performing a CCPP, in which a cue indicated (validly or invalidly) the position of an incoming auditory target. Three different types of blocks with validities of 50%, 64%, and 88%, respectively, were presented. Estimates of prior expectation and surprise were obtained on a trial-by-trial basis from participants’ responses, using a computational model implementing Bayesian learning. These two values were correlated on a trial-by-trial basis with the EEG values in all the electrodes and time bins. Therefore, a Spearman correlation metrics of the relationship between Bayesian parameters and the EEG was obtained. We report that the surprise parameter was able to classify the different validity blocks. Furthermore, the prior expectation parameter showed a significant correlation with the EEG in the cue-target period, in which the Contingent Negative Variation develops. Finally, in the post-target period the surprise parameter showed a significant correlation in the latencies and electrodes in which different event-related potentials are induced. Our results suggest that Bayesian parameters are coded in the EEG signals; and namely, the CNV would be related to prior expectation, while the post-target components P2a, P2, P3a, P3b, and SW would be related to surprise. This study thus provides novel support to the idea that human electrophysiological neural activity may implement a (Bayesian) predictive processing scheme.

## Introduction

Wide consensus is accumulating around the idea that the human brain is a *prediction machine*, which learns statistical regularities in the form of internal generative models and uses the models to continuously generate predictions to guide perception and action (von Helmholtz, 1866; Rao and Ballard, 1999; Friston, 2005, 2010; Doya et al., 2007; Bar, 2009; Pezzulo et al., 2014, 2015, 2017, 2018; Summerfield and de Lange, 2014; Friston et al., 2015, 2016a,b, 2017). We still have scarce evidence on the ways the human brain may implement the computational steps required for Bayesian inference and learning, e.g., the generation of expectations prior to observing stimuli and of surprise or prediction error signals afterward (Egner and Summerfield, 2013; Pouget et al., 2013).

An effective way to understand how the brain may extract and encode statistical regularities during a cognitive task is performing a model-based computational analysis of participants’ behavioral and brain data (Daw, 2009). Previous studies using a model-based methodology have identified a variety of Bayesian parameters to be used as regressors for fMRI or EEG data, which include: *Predictive surprise*, which represents the subjective information content, or surprisal received when an event is observed (Shannon, 1948); *Bayesian surprise*, or the degree of updating in the beliefs after experiencing a new event (Baldi and Itti, 2010); *Prior expectation*, or (the mean of) the expected probability for a given event before the observations (note that in a trial-by-trial analysis, the posterior expectation at some trial T can be considered as the prior expectation at the next trial T+1).

A study employing the Hierarchical Gaussian Filter (HGF; Mathys et al., 2014) for a model-based analysis of a sensory learning task reported that fMRI activity in the visual, supramodal, and midbrain indexed low-level sensory prediction errors, whereas fMRI activity in the basal forebrain indexed higher-level prediction errors (Iglesias et al., 2013). Another study combining electrophysiological and neuroimaging approaches reported signatures of Bayesian inference at multiple hierarchical levels during a social learning task (Diaconescu et al., 2017). The model-based approach has been also widely used in combination to EEG techniques. Indeed, event-related potentials (ERPs) provide high time resolution of neural activity and are particularly suited to investigate dynamical neural phenomena like the coding of prediction errors in Bayesian models. For example, (Kolossa et al., 2013) showed that short and long-term effects of previous targets on P300 amplitude are modeled by means of digital filters with different time constants; see also (Squires et al., 1976; Duncan-Johnson and Donchin, 1977) for other approaches to study the neural signatures of Bayesian computations in ERPs.

Another line of research using a Bayesian approach in combination with ERPs has shown that, in an urn-ball paradigm, three components of the P300 late positive complex – P3a, P3b, and positive Slow Wave – index dissociated different processes of Bayesian inference: the updating of Bayesian surprise (updating of beliefs about hidden states), predictive surprise (the subjective information content received from an observed event) and the updating of predictions of observations (the so-called postdictive surprise) (Kolossa et al., 2015). Finally, another related ERP study using the urn-ball task linked P3a and P3b signals to prior probabilities and likelihoods, respectively (Kopp et al., 2016); see also (Seer et al., 2016). Yet, despite these progresses, there are several aspects of a putative neural coding of Bayesian parameters that remain incompletely understood. In particular, while surprise signals have been studied quite extensively, the relationship of the prior expectation parameter with the EEG signals has not been addressed directly.

The goal of the present study is testing the quantitative relationships between ERPs and the Bayesian estimations of *prior expectation* and *surprise*, on a trial-by-trial basis, by adopting a model-based approach. Specifically, this study investigates the coding of Bayesian parameters in ERP signals during a Central Cue Posner Paradigm (CCPP), in which participants saw centrally presented cues that were either valid (i.e., correctly cued the target) or invalid. The CCPP task is especially compelling, as it induces an expectancy period between the spatial cue and the target, and a surprise after the target is revealed (if unexpected). Critically, cues had different levels of validity in different blocks (50%, 68%, and 86%, respectively); but those needed to be learned during the task – which implies that participant may experience different subjective degrees of surprise during learning. This manipulation permitted us to study the putative coding of two critical parameters of Bayesian inference – *prior expectation* (i.e., a mean prediction derived from a Bayesian model, see below) and *surprise* (i.e., the discrepancy between prediction and evidence) – in trial-by-trial ERP signals, while participants learned the task. Single-trial *prior expectation* and *surprise* parameters were inferred from participants’ behavior using a Bayesian learning model (HGF, Mathys et al., 2011) that was previously validated in a Posner task (Vossel et al., 2014). The *prior expectation* and *surprise* parameters inferred by the model based were then correlated with ERP signals recorded from the same participants and trials.

Following the hypothesis that the brain implements Bayesian computations (aka, Bayesian brain hypothesis), we expected the single-trial EEG amplitude to correlate with the *prior expectation* parameter in the preparatory period (cue-target period) and with the *surprise* parameter in the belief-updating (post-target) period. This result would indicate that ERP signals index key parameters of Bayesian inference, in the proper temporal order (i.e., prior expectations need to be formed first, to be used to derive surprise signals). In keeping with this hypothesis, it has been suggested that the negative slow potential, termed as Contingent Negative Variation (CNV), may be a neural signature for prior probabilities, and related to the updating of beliefs about the relationship of cue and target (Gómez and Flores, 2011). In the post-event period, the P3a, P3b and late positive slow wave component present higher amplitude in invalid than valid trials during CCPP (Mangun and Hillyard, 1991; Arjona et al., 2016), suggesting a higher neural processing of invalid with respect to valid trials. The amplitude of the late positive complex is sensitive to the validity or invalidity of the trial, but also to the validity probability in a given block (Arjona et al., 2016), i.e., the so-called *global probability*, and to the recent stimulus sequence, i.e., the *local probability* (Arjona Valladares et al., 2017; Arjona et al., 2018).

Furthermore, we expected *prior expectation* and *surprise* parameters to be decoded equally well across the three experimental blocks of the CCPP, which have different statistics. This is important, as it would indicate that the brain continuously tracks task statistics, and generates context-specific predictions and surprise signals (i.e., signals that depend on learned task statistics), rather than using a more inflexible, non-Bayesian strategy that simply reacts differentially to invalid versus valid trials (e.g., by elevating EEG signals for invalid trials).

## Materials and Methods

### Subjects

Thirty subjects (15 females and 15 males) between 18 and 35 years of age (mean: 24 years old and SD: 4.22) participated in the experiment. Two of them were eliminated because the EEG was not properly recorded. After computing the *prior expectation* and *surprise* parameters with the HGF model (Mathys et al., 2011), nine subjects who only produced negligible changes in these parameters on a trial-by-trial basis were eliminated from further analysis. The remaining sample of 19 subjects was completely analyzed. The experiments were conducted with the informed and written consent of each subject, following the rules of the Helsinki Convention. The “portal of ética en Biomedicina de la Junta de Andalucía” approved the study.

### Paradigm

**FIGURE 1:**
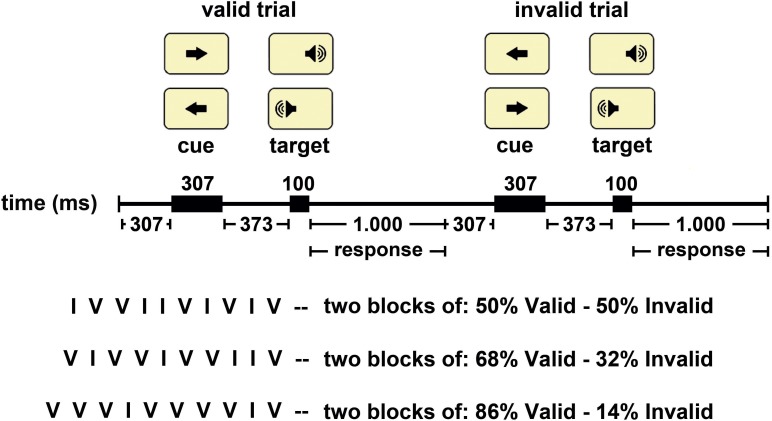
The Central Cue Posner Paradigm used in this study. Representation of a valid and an invalid trial. The arrows indicate the possible location of a target and the position of the target in the current trial is indicated in rectangle. The central arrow (cue) was presented in the center of the screen, and the auditory stimulus (target) was presented monaurally. The temporal sequence of stimulus presentation appears in the middle part of the figure. The inferior part of the figure indicated the three options of validity probability blocks and a typical sequence of valid (V) and invalid (I) trials for each type of block.

The stimuli were presented through E-Prime (2.0), on a computer screen situated at 60 cm of the subjects. The experimental paradigm consisted in a visuo-auditory modified version of the CCPP (Figure 1), with arrow cues appearing at the center of the screen (DELL E773p, Graphic Card NVIDIA GeForce FX 5200, 1152×864 pixels (75 Hz), color and luminance 9,300 K, followed by monaural auditory stimulation (1,000 Hz and 72 db). The central arrow stimulus (S1) was intended to induce spatial orientation, and the monaural auditory stimulus (S2) corresponded to the imperative stimulus. Subjects had to press the right button with the index finger of the right hand if the auditory stimulus appeared in the right ear or the left button with the left index finger if the auditory stimulus was presented in the left ear. The Cedrus (model RB-530) was used as a response device.

The events sequence within a trial was as follows: (I) a central fixation white cross appears for 300 ms; (ii) the S1 is on for 300 ms; (iii) an expectancy period (with the white cross) lasts for 370 ms (therefore, the total S1–S2 period was 670 ms); (iv) the S2 comes on for 100 ms and is randomly presented to the left or right ear, with equal probability (0.5); and (v) the response time is on for 1,000 ms (during this period, the white cross was presented again) (Figure 1).

The experiment consisted of 600 trials divided into six blocks (100 trials per block), and there were three types of blocks: (i) Block validity 50%: in 50% of the trials the S1 points to the correct location where the S2 will appear (valid trials) and in the other 50% the S1 points to the wrong location (invalid trials). (ii) Block validity 68%: in 68% of the trials the S1 points to the correct location where the S2 will appear (valid trials) and in the other 32% the S1 points to the wrong location (invalid trials). (iii) Block validity 86%: in 86% of the trials the S1 points to the correct location where the S2 will appear (valid trials) and in the other 14% the S1 points to the wrong location (invalid trials). The 30 participants were divided into six groups upon a different order of presentation of the blocks (six block orders). Different validity blocks are then counterbalanced, and the possible effect caused by the influence of the previous type of block was canceled. There were 12 training trials.

### EEG Recordings

The EEG was recorded from 32 scalp sites in an extended version of the International 10–20 System, using tin electrodes mounted on an electrode cap (Electrocap). Impedance was maintained below 5 KOhms. Data were recorded in DC using a common average as reference, and they were not filtered. The ground electrode was located on the line between Fpz and Fz. The amplification gain was 20.000, and the data were acquired at a sampling rate of 512 Hz (ASA-lab EEG/ERP system, ANT, Holland). EEG recordings were analyzed with the EEGlab v10.0.0.0b (Delorme and Makeig, 2004) and Matlab R2016a (MathWorks Inc., MA, United States) software packages. To eliminate AC power line interference and blink artifacts in the EEG, an Independent Components Analysis (Groppe et al., 2009) was performed. Criteria for determining these artifactual components were their scalp map distribution, time course and spectral power. These components were discarded, and the EEG signal was reconstructed.

### Behavioral Analysis

The reaction time (RT) and errors of the present experiment were previously published (Arjona et al., 2016). Results showed the typical pattern of cost-benefit of the CCPP, with faster and more accurate responses in *valid* compared to *invalid* trials. See the Supplementary Material for descriptive statistics of the behavioral data.

Here, we re-analyzed the trial-by-trial data, using the HGF: a computational model that implements hierarchical Bayesian inference and learning (Mathys et al., 2011, 2014; Diaconescu et al., 2017). The rationale for using the HGF model is twofold. First, the HGF embodies the hypothesis that the brain uses a (hierarchical) Bayesian scheme to infer task contingencies (e.g., the validity of cues) and to update these hypotheses when it receives novel information (i.e., after each trial). As it uses a Bayesian scheme, the HGF provides an estimate of each participant’s *prior expectation* and *surprise* parameters during the task, on a trial-by-trial basis, by fitting participants’ response times (RS) for valid and invalid trials. Correlating these parameters with EEG signals on a trial-by-trial basis would allow us testing the hypothesis that the brain might perform related Bayesian computations. Second, the HGF was already validated in the context of a CCPP task (Vossel et al., 2014), which provides some confidence in the fact that the theoretical assumptions it makes (e.g., about how participant’s response times can be modeled) are sufficiently realistic in this context.

The HGF receives as input participant’s response time (in milliseconds) for each trial; note that trial order of each participant is preserved. Moreover, each trial has two labels, which indicate whether the trial is valid (i.e., cue and target are congruent) or invalid (i.e., cue and target are incongruent), and whether the response was correct or an error (in this latter case, the trial is ignored). The HGF uses this input to infer participants’ hidden states or beliefs about the task (e.g., about cue validity, see later) that best explain their observed responses, and which vary on a trial-by-trial basis (and can thus be correlated with participants’ trial-by-trial EEG signals). To this aim, the HGF learns the parameters of a so-called *generative model*, i.e., a probabilistic mapping between participants’ hidden beliefs and their responses.

**FIGURE 2:**
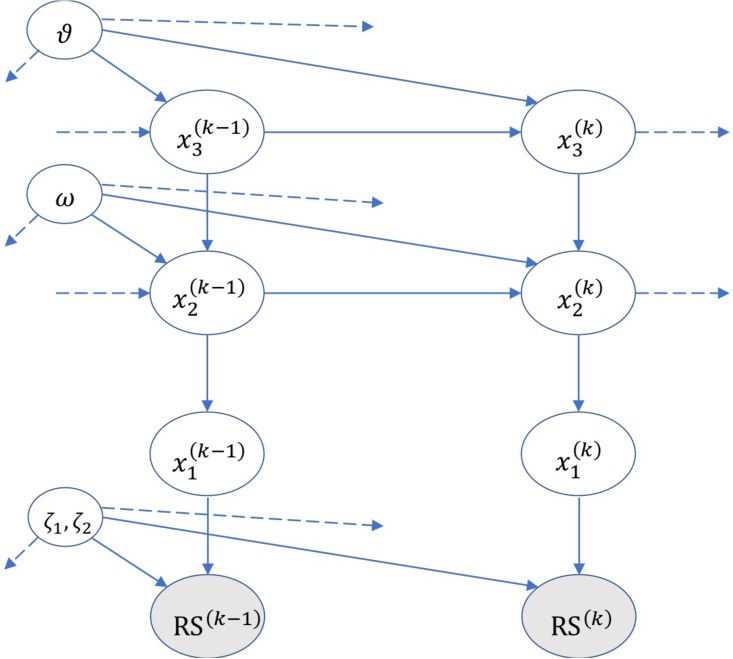
A graphical illustration of the Hierarchical Gaussian Filter (HGF) model of Mathys et al. (2014). The figure shows the hidden states $x_{i}^{\left(k\right)}$ and parameters (ζ, ω, and 𝜗) encoded by the model, and the probabilistic relations (edges) across multiple hierarchical levels and across time (e.g., *k*–1, *k, k*+1). RS denotes the subject’s response. Please see the main text and Mathys et al. (2014) for details on the HGF.

The generative model of HGF includes a hierarchy of hidden states $x_{i}^{\left(k\right)}$, with *i* = 1, 2, 3, denoting the three levels of the model, see Figure 2.

At the first hierarchical level, the model encodes the state $x_{1}^{\left(k\right)}$ whose binary values represent the category (valid or invalid) of the current trial. The probability of the state $x_{1}^{\left(k\right)}$ is conditioned on a second-level real variable $x_{2}^{\left(k\right)}$, according to the following rule:

$$x_{1}^{k}|x_{2}^{k}\sim Bernoulli\left(x_{1}^{k};s\left(x_{2}^{k}\right)\right)$$

where *s*(*x*) = (1 + *e*^-x^)^-1^. Here, the state $x_{2}^{k}$ is interpreted as the ‘tendency’ of the stimulus to be valid. By hypothesis, the value of $x_{2}^{k}$ evolves across trials following a Gaussian random walk and is normally distributed around its value at the previous trial:

$$x_{2}^{k}\;\sim \;N\left(x_{2}^{\left(k-1\right)};\;e^{\left(x_{3}^{\left(k\right)}+\omega \right)}\right)$$

with variance described by the term *exp*($x_{3}^{\left(k\right)}$ + ω), where ω is a measure of the trial-by-trial variability in $x_{2}^{k}$.

At the highest hierarchical level, the state $x_{3}^{k}$ expresses the ‘log-volatility’ (e.g., a rate of change of the statistics) of the environment. (Note that our CCPP task comprises three types of blocks having different cue validities – 50%, 68%, and 86% – hence one can consider that shifting block types entails a form of volatility in the cue validity statistics.) The state $x_{3}^{k}$ is assumed to be normally distributed around its value of the previous trial, with a variance defined by a subject-specific parameter 𝜗, which denotes the variability of the volatility over time (‘meta-volatility’).

$$x_{3}^{k}\sim N\left(x_{3}^{\left(k-1\right)};\;\vartheta \right)$$

The HGF uses Bayesian inference to update all the aforementioned posterior densities (or beliefs) of the variables $x_{1}^{k}$, $x_{2}^{k}$, and $x_{3}^{k}$ on a trial-by-trial basis, by integrating sensory evidence (about cue validity). As full Bayesian inference can be intractable, the HGF uses variational model inversion and a mean field approximation (Mathys et al., 2011; Vossel et al., 2014), to calculate the posterior densities of the variables $x_{i}^{k}$. By denoting the mean and precision (i.e., inverse variance) of $x_{i}^{k}$ as μ and π, respectively, the update of $\mu_{i}^{k}$ at level *i* and trial *k* has a general form (with a slightly different form for *i* = 2), see (Mathys et al., 2011; Vossel et al., 2014):

$$\mu_{i}^{k}-\mu_{i}^{\left(k-1\right)}\propto \frac{{\hat{\pi }}_{i-1}^{\left(k\right)}}{\pi_{i}^{k}}\delta_{i-1}^{\left(k\right)}$$

where the accent (^) designates the expected value predicted before observations and $\delta_{i-1}^{(k)}$ is the prediction error about the input coming from the level below.

The lowest level of the hierarchical generative model of HGF describes how the subjects’ beliefs cause their response times at each trial *k*. The model of (the inverse of) subjects’ response time RS is:

$$RS^{k}=\{\begin{array}{l} \zeta_{1\mathrm{valid}}+\zeta_{2}\;\alpha^{k}\;\;\;\;\;\;\;\;\;\;if\;x_{1}=1\\ \zeta_{1\mathrm{invalid}}+\zeta_{2}\;\left(1-\alpha^{k}\right)\;\;\;\;\;\;\;\;\;\;\;if\;x_{1}=0 \end{array}$$

Note that RS denotes the inverse of response time, not response time, as the former but not the latter is assumed to follow a normal distribution. Furthermore, the response time model is separated for valid and invalid trials, which follow different distributions in CCPP and other tasks (Mathys et al., 2011; Vossel et al., 2014).

The parameters ζ_1valid_, ζ_1invalid_, and ζ_2_ of the RS model are estimated along with all the other parameters of HGF, using subjects’ responses for each trial and the factor α^k^ defined as:

$$\alpha^{k}=\frac{1}{\left(1-log_{2}{\hat{\mu }}_{1}^{\left(k\right)}\right)}$$

Here, -*log*_2_${\hat{\mu }}_{1}^{\mathrm{(k)}}$ is the Shannon surprise for the predicted stimulus ${\hat{\mu }}_{1}^{\mathrm{(k)}}$, defined as the softmax ${\hat{\mu }}_{1}^{\mathrm{(k)}}$ = *s*(${\hat{\mu }}_{2}^{(k)}$ of the belief for the rate of change. Note that, intuitively, α is assumed as an attention factor, which becomes zero with zero surprise.

We used the HGF model to calculate two parameters – *prior expectation* and *surprise* – for each subject (as we estimated ζ, ω, and 𝜗 separately for each subject) and for each trial (as the HGF is sensitive to the order of trials actually experienced by each subject), based on subjects’ response times during the task.

Prior expectation (on $x_{1}^{\left(k\right)}$) corresponds to ${\hat{\mu }}_{1}^{\mathrm{(k)}}$ and is calculated by the HGF model as the mean of the (probabilistic) expectation of the cue validity, for each subject and for each trial. Surprise corresponds to -*log*_2_${\hat{\mu }}_{1}^{\mathrm{(k)}}$ and is calculated by the HGF model as a function of the prior expectation and the actual cue validity at each trial. The term “surprise” refers here to the Shannon (1948) surprise associated with each target stimulus during the experiment. It is worth noting that while in some settings Shannon surprise for a given stimulus can be considered an objective measure and is defined as the negative logarithm of stimulus probability, here it is a subjective measure. This is because participants have to estimate their subjective probability of stimuli, using their own generative model of the task, which is different from subject to subject and depends on the history of previous trials that each subject experienced.

### EEG Analysis

An offline filtering of 0–30 Hz was applied to the EEG. Independent Component Analysis Artifact (Delorme and Makeig, 2004) corrected recordings were averaged off-line using a rejection protocol based on the voltage amplitude: All the epochs for which the EEG exceeded ±100 microvolts in any channel were automatically discarded for ERP analysis. The algebraically linked mastoids were computed off-line and used as a reference for analytical purposes. ERPs were obtained for each subject by averaging the EEG, using the switching-on of the cue and the target as trigger. Two baselines were used to compute the ERPs; (i) for the CNV period was the -100 to 0 ms interval before the cue stimuli; and (ii) for the post-target components N1, P2, P2a, P2p, P3a, P3b, and SW was the -100 to 0 ms interval before the target stimuli.

**FIGURE 3:**
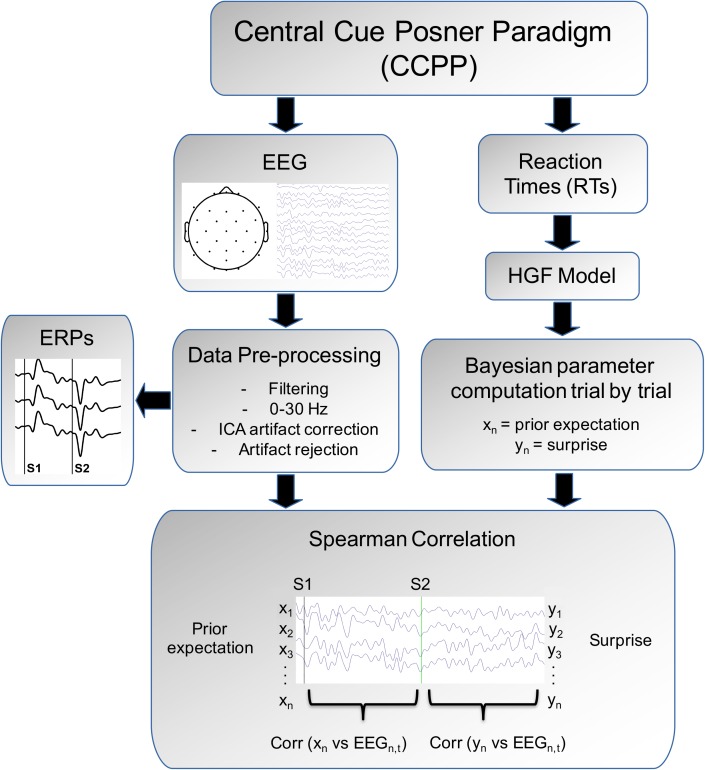
Analysis pipeline. The subject is presented to a Central Cue Posner Paradigm and Reaction times and EEG are collected. From the HGF model we derive the surprise prior expectation (Xn) and surprise parameter (Yn). The EEG, after data pre-processing, permits to obtain the event-related potentials (ERPs) induced by the arrow cue (S1) and by the auditory target (the statistics for the ERPs are described in Arjona et al., 2016). Finally the correlations in a trial-by-trial basis between the HGF parameters (Xn and Yn) with the EEG are obtained. See more details in the “Materials and Methods” section.

To compute the possible relations between the Bayesian parameters (*prior expectation* and *surprise*) inferred by the HGF and the EEG signal, we performed a trial-by-trial correlational analysis (see Figure 3 for the complete pipeline analysis). To test the hypothesis that the *prior expectation* parameter was related to the EEG time window in which Contingent Negative Variation (CNV) develops, the *prior expectation* of individual subjects was correlated with the EEG values in the following manner: for each time bin, electrode and trial, the voltage value was obtained and then Spearman correlated with the prior expectation value. This procedure was used in the period between S1 and S2 and the corresponding pre-cue period [-200 (pre-S1) to 670 (post-S1) ms]. As indicated in Figure 3, the vector of the prior expectation parameter (Xn, a vector with dimensions: number of trials × 1) is correlated with the columns of the matrix EEGn,t (a matrix with dimensions: number of trials × number of time points). The prior expectation computed from the model in a given trial is correlated with EEG values at the next trial, given that the expectation concerns the (validity of the) next stimulus. This computation provides a vector with the correlation of the prior expectation parameter vs. the spontaneous EEG values along time (dimensions: number of time points × 1). For presentation purposes, the grand-average of the individual correlational vectors was computed.

To test the hypothesis that the *surprise* parameter was related to the EEG after appearance of the target, the voltage values in each time bin, electrode and trials were Spearman correlated to the surprise parameter during the post-target period [1,000 (post-S2) ms] and corresponding pre-target period [-200 (pre-S2)]. This procedure was similar to that described for the CNV period (see above), but using the surprise parameter and the post-target time window. For correlations, surprise is aligned with the EEG of the same trial in which is computed, given that surprise represents the subjective information content of an event when is observed (in this case the auditory target) (Shannon, 1948). The correlation analyses between the EEG and the HGF parameters were computed using “Matlab R2016a.”

We analyzed how EEG signals related to Bayesian parameters, by focusing on the same electrodes and time windows in which differences in ERPs for valid and invalid trials were previously obtained (Arjona et al., 2016). The obtained Spearman correlation (between the EEG and the Bayesian estimated parameter) was then used as a metrics for the statistical comparison against the pre-cue or pre-target periods. For the CNV, the relevant time window was 370–670 ms after the cue arrow (S1) and the analyzed electrodes Fz and Cz. For the post-target components, the latencies and electrodes corresponded to N1 (90–120 ms, FC1, FC2, C3, Cz, C4); P2a (153–193 ms; F3, Fz, F4, FC1, FC2); P2p (153–193 ms; CP1, CP2, P3, Pz, P4); P3a (290–340 ms; FC1, FC2, Cz), P3b (380–530 ms; P3, Pz, P4, POz, O1, Oz, O2) and the negative slow wave (SW) (380–530 ms; FP1, FPz, FP2, F3, Fz, F4).

We made an additional effort to find possible significant effects in the component P2 (P2a and P2b are obtained as subtraction of ERPs in invalid from ERPs in valid trials). For assessing possible Bayesian effects of the P2 component, the electrodes Cz, Pz, and POz – which are the electrodes showing the higher amplitude for this component (Arjona et al., 2016) – were selected in the same time window as P2a and P2p (153–193 ms). The mean of the Spearman correlation coefficient was obtained for these latencies and electrodes. The pre-cue and pre-target periods for the Spearman correlation coefficient were obtained for the same electrodes in the time window -200 to 0 ms before the cue (for the CNV analysis) and before the target (for the post-target analysis), respectively.

An independent ANOVA was computed for each component, with the factors time window (pre-cue or pre-target period and time window of a given component as levels) and electrodes. Please notice that Spearman correlation can have positive and negative values, therefore what the statistics is testing is if Spearman correlation (between the EEG and the estimated Bayesian parameter) is significantly different from the interval -200 to 0 ms previous to the cue or to the target, in the time window corresponding to a given ERP component. The Greenhouse–Geisser correction for sphericity was applied when sphericity was not obtained. *T*-test were used as *post hoc* analysis when needed. The ANOVAs were computed using “SPSS_23.”

Finally, the correlations of the single trial EEG values versus the surprise parameter estimated from the HGF model were compared (by means of ANOVA) with the correlations obtained between the EEG and a model that assumes that subjects know the true probabilities of the stimuli. The true probabilities were obtained from the empirical validity probability of each block of trials (e.g., in the 86% validity block the true probability for valid trials was 0.86, and the complementary 0.14 for invalid trials). Then the (Shannon) surprise for the true probability parameter is computed as *S* = -log_2_ *p*. Although subjects cannot calculate the same surprise values (as they do not have access to the true probabilities), we asked if they could approximate it – and whether the surprise values calculated using the true probabilities and/or extracted from the HGF model correlate well with the EEG signal. If the surprise estimated from the HGF model is coded in the EEG, and corresponds to a good estimation of the empirical surprise imposed by the stimuli sequence, the correlations between the HGF surprise and the EEG should be similar to the correlations between the EEG and the surprise estimated from the true probabilities. To obtain the correlations between the single trials EEG and the true probability, we adopted the same approach as shown in Figure 3, but used the surprise estimated from the true probabilities rather than the surprise inferred by the HGF model.

## Results

**FIGURE 4:**
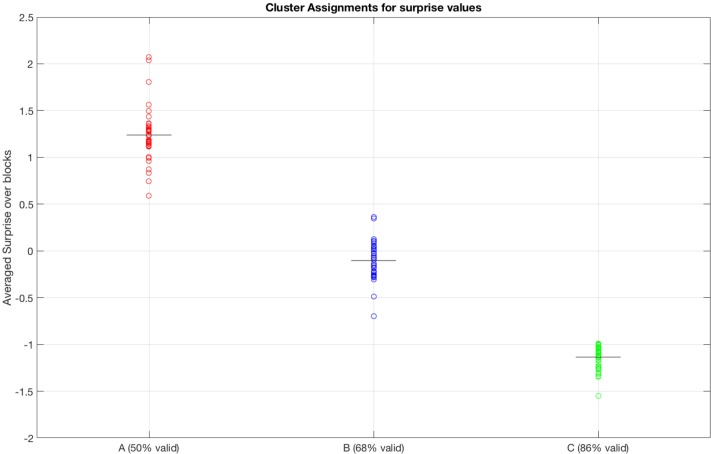
Clustering of blocks from the surprise parameter inferred by the HGF model. A, B, and C represent three blocks with different percentages of cue validity (50%, 68%, and 86%, respectively). Note that the HGF model recovers well the statistically differences between the blocks, by forming three distinct blocks.

We tested whether the surprise parameter inferred by the HGF model (Mathys et al., 2011) was sensitive, and sufficient to discriminate the behavior of participants in different blocks. The results shown in Figure 4 show that the (average) surprises calculated by the HGF model cluster subjects robustly by block, thus indicating that the HGF model provides sensitive parameters. The clustering procedure was applied to the initial 28 subjects.

**FIGURE 5:**
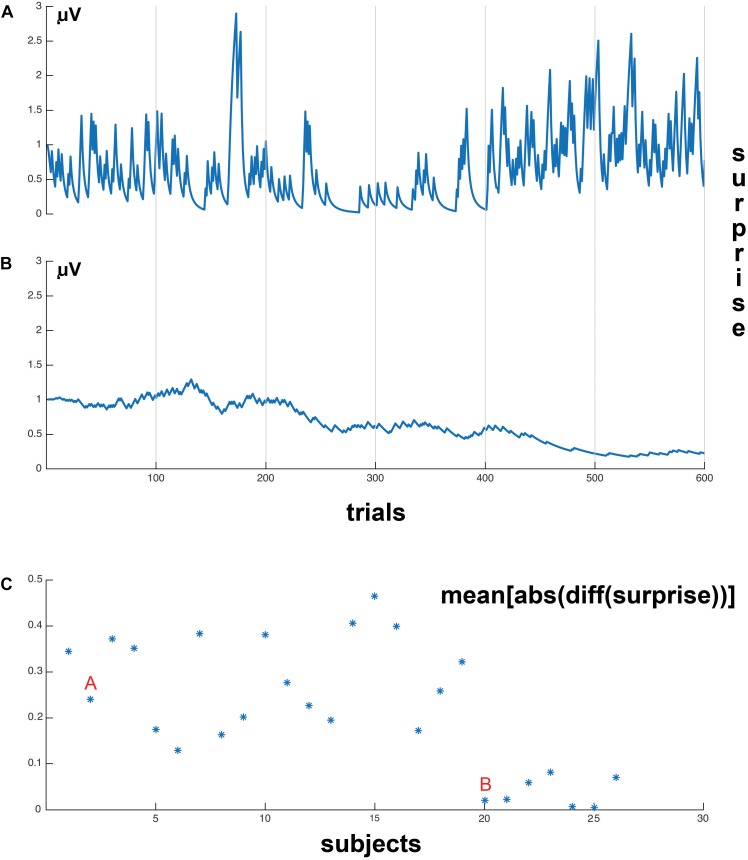
Surprise values, on a trial-by-trial basis. **(A)** Example subject whose surprise values are strongly related to current block validity (which changes every 200 trials) and trial-by-trial changes in validity (sharp peaks of surprise values). **(B)** Example subject whose surprise values are only related to the current block trial validity. **(C)** Represents the mean sensitivity to change for each individual subject, the mean[abs(diff (surprise))] represents the computation method to obtain the mean sensitivity to surprise to the validity or invalidity of the trial in each subject (see section “Materials and Methods”). “A” indicates the cluster of subjects with high sensitivity to invalidity, and “B” the subjects with low sensitivity to invalidity. Please notice that the 19 first subjects presented a much higher sensitivity to changes in trial validity than the seven subjects at the right of the display. This difference in the sensitivity of surprise to validity change permitted to split the sample in two subgroups. The correlational analysis was exclusively applied to the 19 subjects at the left (high sensitivity of surprise to changes in validity).

However, the results of the HGF model show two different trial-by-trial surprise patterns for the participants, which are shown in Figure 5A,B, respectively. The first pattern corresponded to 19 subjects, who showed a big response to surprise in invalid trials and also adjusted surprise in valid trials to the mean surprise of the block (Figure 5A). The second pattern corresponded to seven subjects, who showed small response to Invalid trials, despite they adjusted to the mean surprise of the block of trials (Figure 5B). We quantified these different patterns computing the differential (Matlab function *diff*) with respect to trial order, to obtain a metric of the sensitivity of the subjects with respect to changes in experimental trial sequences (e.g., Valid→Invalid, Invalid→Valid). We computed the absolute value to the differential of surprise and then the mean value across trials of the differential, i.e., a sensitivity to change (defined as mean[abs(diff(surprise))]) for each subject. Figure 5C shows 19 subjects (labeled with an A) with high sensitivity to change and seven subjects (labeled with a B) with a low sensitivity to change. As one of the primary interests of the present study was to correlate post-target EEG with the surprise parameter, we limited the EEG correlation analysis to the 19 subjects with high sensitivity to change. This is because the seven subjects that did not show a clear response to surprise in invalid trials presented a very low surprise variance on a trial-by-trial basis, and this pattern would not permit to obtain significant correlations with the EEG signal.

**FIGURE 6:**
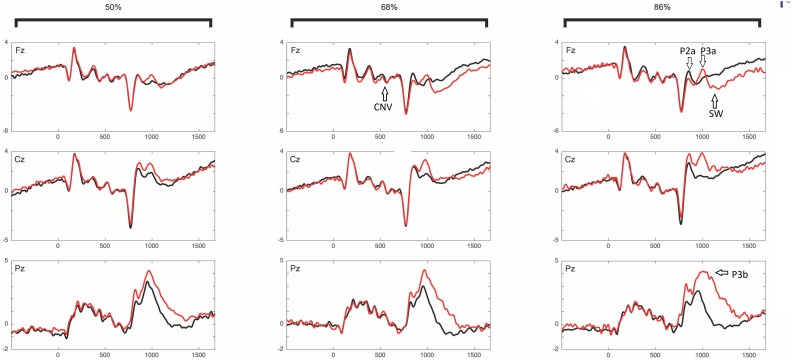
Event-related potentials: CNV, P2a, P3a, P3b, and SW. ERPs are Presented into different validity blocks (50%, 68%, and 86% of validity, respectively), and by showing separately lines for valid (black) and invalid (red) trials within each validity block. See Arjona et al. (2016) for the topographical differences between blocks and validity conditions. The different ERP components in which statistically significant correlations between the EEG and the prior expectancy or surprise parameters were obtained are labeled in the ERPs.

**FIGURE 7:**
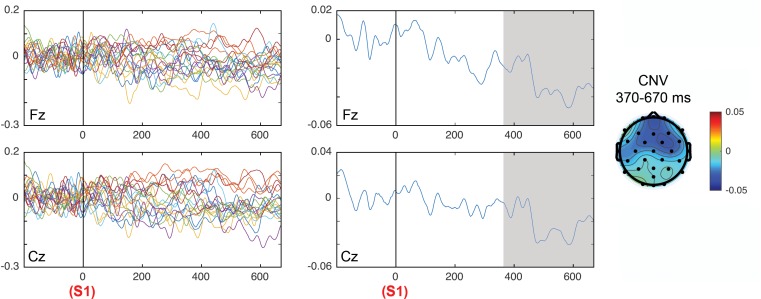
Correlation of the prior expectation parameter with the EEG values in the period between cue (S1) and target (S2), for the electrodes Fz and Cz. The left panels show the correlation in all individual subjects and the middle panel shows the average of correlations. The topography of correlations is displayed in the right panel. The time windows in which the correlations between the EEG and the prior expectation parameter were significant are gray shaded.

Figure 6 shows the ERPs grand average in selected electrodes in the pre-target (preparatory period) and post-target periods of components CNV, P2, P3a, P3b, and SW. Notice the increase of the P3b component in invalid trials (red) with respect to valid trials (black), and in the high validity blocks with respect to low validity blocks. See Arjona et al. (2016) for the topographical differences between blocks and validity conditions. Figure 7 (left panel) shows the Spearman correlation values of single trial EEG voltage values with the *prior expectation* values in the post-S1 period for the 19 subjects, while Figure 7 (middle panel) shows the grand average of the correlation. Notice the increase of negative correlation in this period as time increases. The Figure 7 (right panel) shows the frontal topography of the correlation between EEG and prior expectation in the time window of the CNV component (S1–S2 period). The ANOVA results showed a statistically significant difference of the Spearman correlation between EEG and the prior expectation in the pre-cue time window with respect to the CNV time window; with the time window as a main effect [*F*(1,18) = 5.75, *p* = 0.028; $\eta_{p}^{2}$ = 0.242].

**FIGURE 8:**
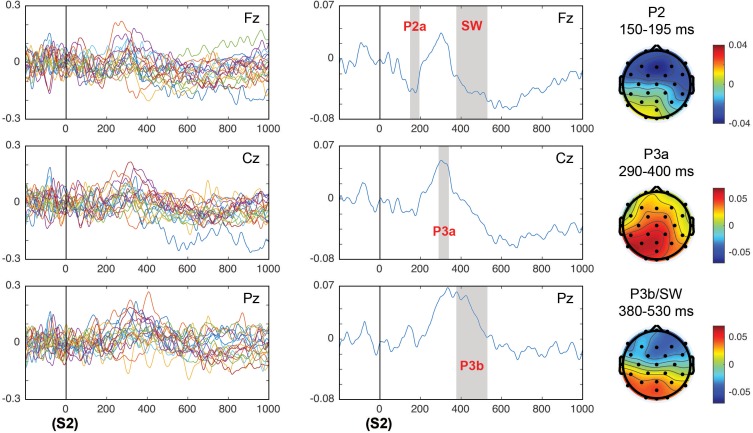
Correlation of the HGF surprise parameter with the EEG values in the post-target (S2) period of the components P2, P3a, and P3b/SW. The left panels show the correlation in all individual subjects and the middle panels show the average of correlations. The topography of correlations for components P2, P3, and P3b/SW is displayed in the right panels. The time windows in which the correlations between the EEG and the surprise parameter were significant are gray shaded, indicating the ERP component that corresponded to this particular time window.

Figure 8 shows the correlation values of single trial EEG voltage values with the *surprise* parameter in individual subjects (Figure 8, left panel) and in the grand average (Figure 8, middle panel). The topographical representation of the correlations for the time windows of the different ERPs analyzed are displayed. For the correlation of the EEG values in the time windows of the different post-target ERPs with the surprise parameter, main effects of the time window factor were obtained in the P2a *F*(1,18) = 15.63, *p* < 0.001; $\eta_{p}^{2}$ = 0.465 in frontal sites (due to a negative correlation between surprise and voltage), in the P3a latency a *F*(1,18) = 6.24, *p* = 0.022; $\eta_{p}^{2}$ = 0.257 with a positive correlation central-posterior topography, P3b *F*(1,18) = 9.68, *p* = 0.006; $\eta_{p}^{2}$ = 0.35 showing a posterior positive correlation topography, and the negative SW *F*(1,18) = 5.59, *p* = 0.029; $\eta_{p}^{2}$ = 0.237 with a negative anterior topography. The central-posterior topography of the correlation in the P3a latency suggests that this correlation indicates a mixed effect of the P3a and P3b components. However, in the late latencies (Figure 8, right panel) the displayed topography corresponded to the typical posterior topography of P3b and the anterior negative SW. Additionally, there was an interaction between the effects of the time window and electrodes factors in the P2 component [*F*(1.52,27.41) = 8.04, *p* = 0.004; $\eta_{p}^{2}$ = 0.309]. The correlation metrics for the P2 component was statistically significant only in the electrode Cz (*p* = 0.049).

**FIGURE 9:**
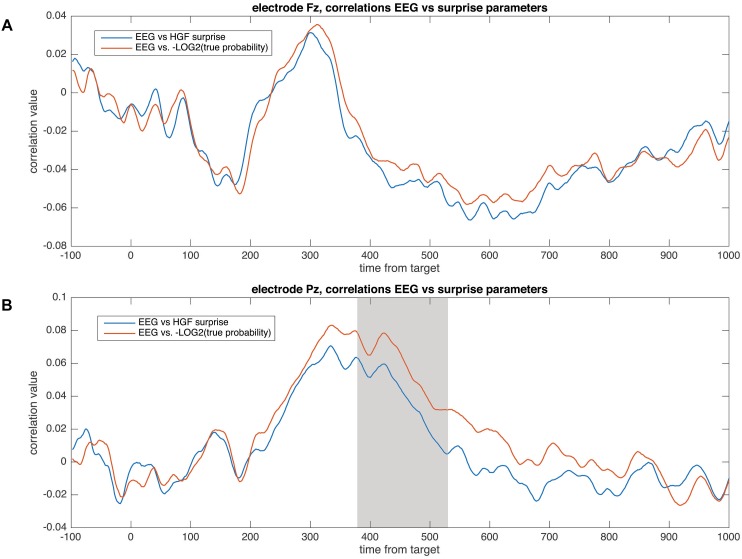
Correlation values between the EEG and the surprise parameters computed from the HGF model and from the true probability for each block, for the electrodes Fz **(A)** and Pz **(B)**. The gray shaded area corresponds to the time window in which the correlations were significantly different (in the P3b time window; see the “Results” section).

In those components in which the correlations of the EEG versus the surprise parameter were significant, an ANOVA comparison with the correlations of the EEG versus the true stimuli probability was computed. Only the P3b component presented a statistically significant difference between these correlations (EEG vs. surprise) due to a higher correlation of the surprise computed from the true probability parameter with respect to the surprise computed from the HGF model [*F*(1,18) = 5.8, *p* = 0.027; $\eta_{p}^{2}$ = 0.244] (Figure 9).

## Discussion

Our results show that, when analyzed on a trial-by-trial bases, the EEG signal collected while participants performed a Central Cue Posner task index critical parameters of Bayesian inference-*prior expectation* and *surprise* – as derived from a computational (Bayesian) learning model, the HGF. Importantly, our analysis revealed that the intervals during which statistically significant results between valid and invalid trials could be decoded in the EEG signal are compatible with the time course of Bayesian inference, with *prior expectation* signals decoded after the cue period, during the CNV period (Figure 6, 7), and *surprise* signals occurring after the target, at the critical latency of P300 (Figure 7, 8), which has long been associated to processes of surprise and information gain (Donchin, 1981; Kolossa et al., 2013). Interestingly, the predictive capacity of the surprise parameter inferred by the HGF model was the same as the predictive power of the surprise computed as if the subjects were aware of the true probabilities of the experiment, except for the time window of P3b. This result suggests that the surprise inferred by the HGF model approximates a perfect knowledge of the empirical stimuli probabilities imposed by the experimental design; but yet some aspects of the coding of surprise may not be fully considered in the HGF model.

Furthermore, our paradigm using three different blocks with different levels of cue validity illustrates that Bayesian parameters can be decoded reliably even when task statistics change. The Bayesian parameters extracted from the computational model appear to track the changing statistics of cue validity, rather than simply reflecting a fixed strategy of response to invalid versus valid trials. This result is in keeping with the idea that the brain continuously performs statistical learning and inference. Interestingly, the tracking of the EEG correlations with the Surprise parameter have been obtained in the same latencies and electrodes in which significant differences are obtained in ERPs between valid and invalid trials (P2, p3a, P3b and negative slow wave) (Arjona et al., 2016). Similarly, the prior expectation parameter presented EEG correlations during the CNV period. Previous studies have shown that CNVs are modifiable by the outcome of previous trial – i.e., they increase amplitude after a valid trial (Arjona et al., 2014) and reduce amplitude after an invalid trial (Arjona Valladares et al., 2017; Arjona et al., 2018). This body of evidence suggests that CNV may index the expectation of the next event at the neural level.

The HGF model was able to model the saccadic latencies from a CCPP (Vossel et al., 2014). The authors of the study showed that a full HGF model, which takes into account the volatility of the environment, approximated saccadic response times better than alternative (i.e., incomplete) models. In their report, the *surprise* parameter extracted by the full HGF model (but not the incomplete models) was able to classify the different blocks of trials – as an increase in trial validity was accompanied by a reduction in Shannon surprise. It is also worth noticing that, similar to the results we report here, in the Vossel et al. (2014) study only a portion of the participants (19 out of 28) showed a clear trial-by-trial change of surprise to invalid trials.

Here, we significantly advance previous findings about the neural encoding of Bayesian parameters, by reporting a significant correlation of the CNV period of EEG with the expectation parameter extracted from the HGF model. This result is consistent with the line of evidence suggesting that CNV may be a marker of top-down expectation in sensory processing (Chennu et al., 2013). The CNV has been associated to expectation in psychophysiological research. Specifically, when a warning informative stimulus (S1) about the characteristics of a second stimulus (S2) is presented, the preparation for S2 induces a change in cortical activity, recorded as a CNV component (Rockstroh, 1982). It has been shown that this preparation induces not only the activation of motor cortex but also the sensory task-specific cortices (Brunia, 1999; Gómez et al., 2003). The CNV would thus correspond to the neural signature of the activation of the task-set neural related areas, by activating areas needed for the subsequent processing of the S2 stimulus (Gómez et al., 2004). CNV localization studies showed that CNV would be composed of different sub-components: An early component that would ignite the preparatory anticipatory properties of CNV, located in anterior cingulate cortex and supplementary motor cortex; a component related to the activation of the motor and sensory cortices needed for the execution of the task; and a Working memory and/or executive attention component, located in dorsolateral prefrontal-cortex and parietal areas (reviewed in Gómez and Flores, 2011).

Single neurons recordings in animals and fMRI studies in humans (Requin et al., 1990; Luck et al., 1997; Kastner et al., 1999) support the tonic activation of the frontal, striate and extra-striate cortices during preparatory periods while visual stimulation is delivered. Therefore, the possible microscopic neural bases of CNV would be in the tonic depolarization of cortical apical dendrites that would increase the neuronal excitability facilitating that firing threshold can be reached in the pre-activated areas (Rockstroh, 1982). It has been proposed that this tonic change in the neural activity baseline would be related at a psychological level with expectation values for a given target (Egner and Summerfield, 2013). The significant correlation of the EEG during the CNV period with the (Bayesian) expectation extracted from the HGF gives a quantitative support to the suggestions that CNV is in fact coding for the quantitative level of expectation of next target (Gómez and Flores, 2011). The correlation of EEG vs. expectation in the CNV period was topographically located in frontal areas, supporting the idea that fronto-striatal circuits are concerned with Bayesian estimation (reviewed in Kopp et al., 2016). The proposal of a CNV coding for the expectation parameter agrees with the study by Bennett et al. (2015), which using a reward anticipation task and recording the so-called Slow Preceding Negativity (a negativity similar to CNV and anticipating rewards) showed a significant regression between the Slow Preceding Negativity and the entropy of the beliefs distribution. The regression showed a decrease in the amplitude of the Negativity with the increase of beliefs entropy, suggesting that a greater certitude about the amount of reward was coded by an increase in amplitude of this anticipatory negative wave.

During the post-target period there was a correlation of the surprise parameter with p2a/P2 component, the late positive complex (covering P3a, p3b and progressing to the late slow positivity) and with the negative frontal SW. P2a would be considered similar to the visual Frontal Selection Positivity, related to task-relevant stimuli processing in the transition from the selection of relevant features to the selection of responses (Kenemans et al., 1993; Makeig et al., 1999; Potts et al., 2004). The time window of P2a is the first time window in which the surprise parameter is significantly correlated to the surprise. The correlation is inverse; i.e., P2a would have higher amplitude with low surprise. This suggests that P2a may be related to confirmatory outcomes of prior expectations, facilitating the selection of adequate responses. This early response to confirmation (earliest than the positive correlations with surprise in the subsequent P3a and P3b) suggests that confirmation of prior expectations relies on frontal sites.

Previous studies have shown the coding of Bayesian parameters in the late positive complex, with Bayesian surprise being coded by P3a and predictive or Shannon surprise coded by the P3b component (Kolossa et al., 2015; Kopp et al., 2016; Seer et al., 2016). As discussed above, the surprise computed through the HGF model is a “subjective” (Bayesian) measure: it comes from a generative model, whose participant-specific parameters are estimated using the participant responses, rather than from an “objective” knowledge of task contingencies. We found this surprise parameter to be correlated with the voltage latencies of P3a and P3b components. During the latency of P3a, the correlation maps have a central and posterior topography, showing a mixed contribution of P3a and P3b. However, the electrodes in which P3a is usually recorded show a significant positive correlation, indicating that P3a would be related to the cognitive processing of the surprise parameter (Kolossa et al., 2015; Kopp et al., 2016; Seer et al., 2016; Higashi et al., 2017). This correlation was prolonged to the latencies of posterior P3b, suggesting that the neural activities related to generation of P3b would also be related to surprise computation. This suggests that surprise may not be computed in a single step, or that its neural trace should be distributed to different areas to produce their cognitive effects.

The EEG data reported here were previously analyzed using a pure psychophysiological approach, with a focus on couples of trials (e.g., the differences between valid–valid versus invalid–valid trials) (Arjona et al., 2016). Here, we extend these previous findings, by using the HGF to study how EEG responses vary as a function of the full history, rather than just the previous trial. Interestingly, the previous reports using the present EEG data found an effect of the global probability (Arjona et al., 2016) and of local probability (Arjona et al., 2018). A lack of interaction between these two levels permitted to suggest independent neural networks for the processing of local (short-term) and global (long-term) probabilities (Arjona et al., 2018), similar to the differential coding of short-term and long-term memory in distinct brain areas. The results reported here did not speak to a separation between local and global probabilities, since the HGF model integrates information across all trials, in a Bayesian way. Therefore, the HGF approach cannot distinguish whether posteriors related to global versus local probabilities are computed independently (Squires et al., 1976; Kolossa et al., 2013; Arjona Valladares et al., 2017; Arjona et al., 2018). The fact that a few subjects presented a surprise value typical of the block, but not phasic changes in surprise in invalid trials (while most of the subjects presented these tonic and phasic changes) suggests that short and long-term effect of estimating the prior probabilities of events could be performed in an independent manner.

The negative frontal slow wave presented a significant correlation with surprise. This component appears in the same time window than P3b and the positive slow wave, but has a different neural origin (Løvstad et al., 2012). From a psychophysiological perspective, it has been proposed that this negative slow wave would represent the reorientation effort after distracters, similarly to the so-called reorientation negativity (Wetzel and Schröger, 2014). Its negative correlation with the surprise parameter would indicate that an unpredictable stimulus would induce the activation of the frontal neural network related to reorientation of attention to the next most probable event.

In sum, we found a reliable trial-by-trial correlation between *prior expectation* and *surprise* parameters extracted by the HGF on the basis of behavioral data collected in a Central Cue Posner task, and EEG signals collected from the same participants in the same trials. Our results contribute to a growing literature showing that the human brain updates critical parameters of Bayesian inference continuously during the task, using principles of statistical learning (that are implicit in how HGF updates its parameters). Specifically, the CNV period would be related to the coding of priors, the P2a component with the confirmation of priors, while P3a and P3b would be related to the coding of trial surprise.

## Ethics Statement

The “portal of ética en Biomedicina de la Junta de Andalucía” approved the study.

## Author Contributions

All the authors designed the study, interpreted the data, and contributed to writing the document. CG, FD, and DM conducted the data analyses.

## Conflict of Interest Statement

The authors declare that the research was conducted in the absence of any commercial or financial relationships that could be construed as a potential conflict of interest.

## References

[B1] Arjona A. Escudero M. Gómez C. M. (2014). Updating of attentional and premotor allocation resources as function of previous trial outcome. *Sci. Rep.* 4:4526 10.1038/srep04526 PMC3970123 24681570

[B2] Arjona A. Escudero M. Gómez C. M. (2016). Cue validity probability influences neural processing of targets. *Biol. Psychol.* 119 171–183. 27430935 10.1016/j.biopsycho.2016.07.001

[B3] Arjona A. Rodríguez E. Morales M. Gómez C. M. (2018). The influence of the global/local probability effect on the neural processing of cues and targets. a functional systems approach. *Int. J. Psychophysiol. Off. J. Int. Organ. Psychophysiol.* 134 52–61. 10.1016/j.ijpsycho.2018.10.005 30342061

[B4] Arjona Valladares A. Gómez González J. Gómez C. M. (2017). Event related potentials changes associated with the processing of auditory valid and invalid targets as a function of previous trial validity in a Posner’s paradigm. *Neurosci. Res.* 115 37–43. 10.1016/j.neures.2016.09.006 27713025

[B5] Baldi P. Itti L. (2010). Of bits and wows: a bayesian theory of surprise with applications to attention. *Neural Netw. Off. J. Int. Neural Netw. Soc.* 23 649–666. 10.1016/j.neunet.2009.12.007 PMC2860069 20080025

[B6] Bar M. (2009). The proactive brain: memory for predictions. *Philos. Trans. R. Soc. Lond. B Biol. Sci.* 364 1235–1243. 10.1098/rstb.2008.0310 19528004 PMC2666710

[B7] Bennett D. Murawski C. Bode S. (2015). Single-trial event-related potential correlates of belief updating(1,2,3). *eNeuro* 2 ENEURO.76–ENEURO.15 10.1523/ENEURO.0076-15.2015 PMC4606160 26473170

[B8] Brunia C. H. M. (1999). Neural aspects of anticipatory behavior. *Acta Psychol.* 101 213–242. 10.1016/S0001-6918(99)00006-2 10344186

[B9] Chennu S. Noreika V. Gueorguiev D. Blenkmann A. Kochen S. Ibáñez A. (2013). Expectation and attention in hierarchical auditory prediction. *J. Neurosci.* 33 11194–11205. 10.1523/JNEUROSCI.0114-13.2013 23825422 PMC3718380

[B10] Daw N. D. (2009). *Trial-by-trial Data Analysis Using Computational Models.* Oxford: Oxford University Press.

[B11] Delorme A. Makeig S. (2004). EEGLAB: an open source toolbox for analysis of single-trial EEG dynamics including independent component analysis. *J. Neurosci. Methods* 134 9–21. 15102499 10.1016/j.jneumeth.2003.10.009

[B12] Diaconescu A. O. Litvak V. Mathys C. Kasper L. Friston K. J. Stephan K. E. (2017). A computational hierarchy in human cortex. *ArXiv*

[B13] Donchin E. (1981). Surprise!… Surprise? *Psychophysiology* 18 493–513. 10.1111/j.1469-8986.1981.tb01815.x 7280146

[B14] Doya K. Ishii S. Pouget A. Rao R. P. N. (2007). *Bayesian Brain: Probabilistic Approaches to Neural Coding.* Cambridge: The MIT Press.

[B15] Duncan-Johnson C. C. Donchin E. (1977). On quantifying surprise: the variation of event-related potentials with subjective probability. *Psychophysiology* 14 456–467. 905483 10.1111/j.1469-8986.1977.tb01312.x

[B16] Egner T. Summerfield C. (2013). Grounding predictive coding models in empirical neuroscience research. *Behav. Brain Sci.* 36 210–211. 10.1017/S0140525X1200218X 23663509

[B17] Friston K. (2005). A theory of cortical responses. *Philos. Trans. R. Soc. Lond. B Biol. Sci.* 360 815–836. 10.1098/rstb.2005.1622 15937014 PMC1569488

[B18] Friston K. (2010). The free-energy principle: a unified brain theory? *Nat. Rev. Neurosci.* 11 127–138. 10.1038/nrn2787 20068583

[B19] Friston K. FitzGerald T. Rigoli F. Schwartenbeck P. O’Doherty J. Pezzulo G. (2016a). Active inference and learning. *Neurosci. Biobehav. Rev.* 68 862–879. 10.1016/j.neubiorev.2016.06.022 27375276 PMC5167251

[B20] Friston K. FitzGerald T. Rigoli F. Schwartenbeck P. Pezzulo G. (2016b). Active inference: a process theory. *Neural Comput.* 29 1–49. 10.1162/NECO_a_00912 27870614

[B21] Friston K. J. Lin M. Frith C. D. Pezzulo G. Hobson J. Ondobaka S. (2017). Active inference, curiosity and insight. *Neural. Comput.* 29 2633–2683. 10.1162/neco_a_00999 28777724

[B22] Friston K. Rigoli F. Ognibene D. Mathys C. Fitzgerald T. Pezzulo G. (2015). Active inference and epistemic value. *Cogn. Neurosci.* 6 187–214. 10.1080/17588928.2015.1020053 25689102

[B23] Gómez C. M. Fernández A. Maestú F. Amo C. González-Rosa J. J. Vaquero E. (2004). Task-specific sensory and motor preparatory activation revealed by contingent magnetic variation. *Brain Res. Cogn. Brain Res.* 21 59–68. 10.1016/j.cogbrainres.2004.05.005 15325413

[B24] Gómez C. M. Flores A. (2011). A neurophysiological evaluation of a cognitive cycle in humans. *Neurosci. Biobehav. Rev.* 35 452–461. 10.1016/j.neubiorev.2010.05.005 20685362

[B25] Gómez C. M. Marco J. Grau C. (2003). Preparatory visuo-motor cortical network of the contingent negative variation estimated by current density. *NeuroImage* 20 216–224. 10.1016/S1053-8119(03)00295-7 14527582

[B26] Groppe D. M. Makeig S. Kutas M. (2009). Identifying reliable independent components via split-half comparisons. *Neuroimage* 45 1199–1211. 10.1016/j.neuroimage.2008.12.038 19162199 PMC3062525

[B27] Higashi H. Minami T. Nakauchi S. (2017). Variation in event-related potentials by state transitions. *Front. Hum. Neurosci.* 11:75 10.3389/fnhum.2017.00075 PMC5326784 28289380

[B28] Iglesias S. Mathys C. Brodersen K. H. Kasper L. Piccirelli M. den Ouden H. E. M. (2013). Hierarchical prediction errors in midbrain and basal forebrain during sensory learning. *Neuron* 80 519–530. 10.1016/j.neuron.2013.09.009 24139048

[B29] Kastner S. Pinsk M. A. De Weerd P. Desimone R. Ungerleider L. G. (1999). Increased activity in human visual cortex during directed attention in the absence of visual stimulation. *Neuron* 22 751–761. 10230795 10.1016/s0896-6273(00)80734-5

[B30] Kenemans J. L. Kok A. Smulders F. T. (1993). Event-related potentials to conjunctions of spatial frequency and orientation as a function of stimulus parameters and response requirements. *Electroencephalogr. Clin. Neurophysiol.* 88 51–63. 7681391 10.1016/0168-5597(93)90028-n

[B31] Kolossa A. Fingscheidt T. Wessel K. Kopp B. (2013). A model-based approach to trial-by-trial p300 amplitude fluctuations. *Front. Hum. Neurosci.* 6:359 10.3389/fnhum.2012.00359 PMC3567611 23404628

[B32] Kolossa A. Kopp B. Fingscheidt T. (2015). A computational analysis of the neural bases of Bayesian inference. *NeuroImage* 106 222–237. 10.1016/j.neuroimage.2014.11.007 25462794

[B33] Kopp B. Seer C. Lange F. Kluytmans A. Kolossa A. Fingscheidt T. (2016). P300 amplitude variations, prior probabilities, and likelihoods: A Bayesian ERP study. *Cogn. Affect. Behav. Neurosci.* 16 911–928. 10.3758/s13415-016-0442-3 27406085

[B34] Løvstad M. Funderud I. Lindgren M. Endestad T. Due-Tønnessen P. Meling T. (2012). Contribution of subregions of human frontal cortex to novelty processing. *J. Cogn. Neurosci.* 24 378–395. 10.1162/jocn_a_00099 21812562 PMC4090805

[B35] Luck S. J. Chelazzi L. Hillyard S. A. Desimone R. (1997). Neural mechanisms of spatial selective attention in areas V1, V2, and V4 of macaque visual cortex. *J. Neurophysiol.* 77 24–42. 9120566 10.1152/jn.1997.77.1.24

[B36] Makeig S. Westerfield M. Jung T.-P. Covington J. Townsend J. Sejnowski T. J. (1999). Functionally independent components of the late positive event-related potential during visual spatial attention. *J. Neurosci.* 19 2665–2680. 10087080 10.1523/JNEUROSCI.19-07-02665.1999 PMC6786079

[B37] Mangun G. R. Hillyard S. A. (1991). Modulations of sensory-evoked brain potentials indicate changes in perceptual processing during visual-spatial priming. *J. Exp. Psychol. Hum. Percept. Perform.* 17 1057–1074. 1837297 10.1037//0096-1523.17.4.1057

[B38] Mathys C. Daunizeau J. Friston K. J. Stephan K. E. (2011). A bayesian foundation for individual learning under uncertainty. *Front. Hum. Neurosci.* 5:39 10.3389/fnhum.2011.00039 PMC3096853 21629826

[B39] Mathys C. D. Lomakina E. I. Daunizeau J. Iglesias S. Brodersen K. H. Friston K. J. (2014). Uncertainty in perception and the hierarchical gaussian filter. *Front. Hum. Neurosci.* 8:825 10.3389/fnhum.2014.00825 PMC4237059 25477800

[B40] Pezzulo G. Kemere C. van der Meer M. (2017). Internally generated hippocampal sequences as a vantage point to probe future-oriented cognition. *Ann. N. Y. Acad. Sci.* 1396 144–165. 10.1111/nyas.13329 28548460

[B41] Pezzulo G. Rigoli F. Friston K. J. (2015). Active Inference, homeostatic regulation and adaptive behavioural control. *Prog. Neurobiol.* 136 17–35. 10.1016/j.pneurobio.2015.09.001 PMC4779150 26365173

[B42] Pezzulo G. Rigoli F. Friston K. J. (2018). Hierarchical active inference: a theory of motivated control. *Trends Cogn. Sci.* 22 294–306. 10.1016/j.tics.2018.01.009 29475638 PMC5870049

[B43] Pezzulo G. van der Meer M. A. A. Lansink C. S. Pennartz C. M. A. (2014). Internally generated sequences in learning and executing goal-directed behavior. *Trends Cogn. Sci.* 18 647–657. 10.1016/j.tics.2014.06.011 25156191

[B44] Potts G. F. Patel S. H. Azzam P. N. (2004). Impact of instructed relevance on the visual ERP. *Int. J. Psychophysiol.* 52 197–209. 15050377 10.1016/j.ijpsycho.2003.10.005

[B45] Pouget A. Beck J. M. Ma W. J. Latham P. E. (2013). Probabilistic brains: knowns and unknowns. *Nat. Neurosci.* 16 1170–1178. 10.1038/nn.3495 23955561 PMC4487650

[B46] Rao R. P. Ballard D. H. (1999). Predictive coding in the visual cortex: a functional interpretation of some extra-classical receptive-field effects. *Nat. Neurosci.* 2 79–87. 10.1038/4580 10195184

[B47] Requin J. Lecas J. C. Vitton N. (1990). A comparison of preparation-related neuronal activity changes in the prefrontal, premotor, primary motor and posterior parietal areas of the monkey cortex: preliminary results. *Neurosci. Lett.* 111 151–156. 2110634 10.1016/0304-3940(90)90360-l

[B48] Rockstroh B. (1982). *Slow Brain Potentials and Behavior.* Munich: Urban & Schwarzenberg.

[B49] Seer C. Lange F. Boos M. Dengler R. Kopp B. (2016). Prior probabilities modulate cortical surprise responses: a study of event-related potentials. *Brain Cogn.* 106 78–89. 10.1016/j.bandc.2016.04.011 27266394

[B50] Shannon C. E. (1948). A mathematical theory of communication. *Bell Syst. Tech. J.* 27 379–423. 10.1002/j.1538-7305.1948.tb01338.x

[B51] Squires K. C. Wickens C. Squires N. K. Donchin E. (1976). The effect of stimulus sequence on the waveform of the cortical event-related potential. *Science* 193 1142–1146. 959831 10.1126/science.959831

[B52] Summerfield C. de Lange F. P. (2014). Expectation in perceptual decision making: neural and computational mechanisms. *Nat. Rev. Neurosci.* 15 745–756. 10.1038/nrn3838 25315388

[B53] von Helmholtz H. (1866). “Concerning the perceptions in general,” in *Treatise on Physiological Optics*, ed. Southall J. P. C. (New York, NY: Dover).

[B54] Vossel S. Mathys C. Daunizeau J. Bauer M. Driver J. Friston K. J. (2014). Spatial attention, precision, and Bayesian inference: a study of saccadic response speed. *Cereb. Cortex N. Y. N.* 1991 1436–1450. 10.1093/cercor/bhs418 PMC4014178 23322402

[B55] Wetzel N. Schröger E. (2014). On the development of auditory distraction: A review. *PsyCh. J.* 3 72–91. 10.1002/pchj.49 26271640

