# Supplementary material for: Tracking the Time Course of Bayesian Inference With Event-Related Potentials:A Study Using the Central Cue Posner Paradigm
Source: Front Psychol. 2019 Jun 19;10:1424. doi: 10.3389/fpsyg.2019.01424 (PMC6593096; doi:10.3389/fpsyg.2019.01424)
Supplement: Supplementary file 1 [file Table_1.pdf]

Supplementary information for:

Tracking the time course of Bayesian inference with Event Related Potentials: a study using the central cue Posner paradigm

Carlos M. Gómez, Antonio Arjona, Francesco Donnarumma, Domenico Maisto, Elena I. Rodríguez-Martínez, Giovanni Pezzulo (correspondence: giovanni.pezzulo@istc.cnr.it)

The behavioural CCPP study was conducted on 30 participants. Data on response time (in ms), block validity (50%, 68%, 86%), trial validity (valid, invalid), and response (correct, errors, anticipations, omissions) are provided as separate .csv files, for each subject.

The 19 participants included in our model-based EEG analyses are: 1, 2, 3, 4, 7, 11, 12, 13, 14, 16, 18, 19, 21, 22, 25, 26, 28, 29, 30.

The table below provides descriptive statistics of the behavioural data of the 19 participants.

Trial type	Mean RT (ms)	St. Dev. RT (ms)	Incorrect responses (%)	Anticipations (%)	Omissions (%)
**50% valid**	376.49	141.06	0.16	0.39	0.19
**50% invalid**	404.45	153.18	1.31	0.60	0.41
**68% valid**	351.04	119.18	0.21	0.63	0.19
**68% invalid**	395.41	132.58	1.06	0.32	0.02
**86% valid**	338.82	117.43	0.06	1.05	0.32
**86% invalid**	393.91	133.29	0.68	0.16	0.13
